# Annotations

**Published:** 1889-08-03

**Authors:** 


### ANNOTATIONS.

**Combining Clerks.**

Several letters, written by clerks, have appeared in a morning contemporary, in which the condition and prospects of that meritorious but long-suffering body of men are set forth with considerable feeling. Everybody thinks himself entitled to pity and patronise clerks as a class because they are in the anomolous position of being neither working men with a trade, master tradesmen, nor professional men, but merely men who can write a decent hand, and who can usually be dispensed with at a week's notice without any injury to the business about which they may be employed. There is a strong prejudice, too, among employers in favour of young clerks, as against those who are middle-aged or old. It is felt that whilst young men may be "ordered about" without much compunction, middle-aged or oldish men cannot so easily be treated in an off-hand manner; or at least the average employer, being "a man and a brother," feels constrained to show them a little more consideration. That is sometimes more than he has time for, and so he learns to prefer as a clerk a youth who can be told without ceremony to do this, and he doeth it; or to go there and he goeth, without doubt or question. It is to be feared, too, that there are not only more clerks than there is employment for, but that even those who are still young could more than fill the vacancies. This state of things has now been in existence a long time, and the inevitable result has followed. Clerks have gone down in price in the market and they have consequently gone down in value as men. They are probably the least independent-minded and self-respecting of all classes of Britons. They are also the most helpless and hopeless when thrown out of employment. One clerk, writing to the *Daily News*, speaks of himself as "elderly," although he is not yet forty years of age. At an ordinary business the man would only be approaching his best at forty. As a clerk, he seems to be coming to the end of his career. This is ridiculous. That particular clerk recommends combination; and, undoubtedly, if clerks are still to exist as a class, they must combine for mutual help and defence. By combining they can afford to risk the loss of a situation if it should become intolerable; they can prevent too great a reduction of salaries; they can prepare for possible sickness; they can provide against inevitable old age. As things are at present, they are in a deplorable plight. All that is most marked in the English character seems to be crushed out of them. Many of them are no longer men, but conveniences and stop-gaps whom everybody can bundle about at his pleasure. This ought not to be the case with any class of Englishmen. It is not only ruinous for them, but also for their employers and for the State. A state is what all its citizens are. If they are reduced below the level of a free-minded and honourable manhood, the State can never be either strong, or safe, or worthy. This is not a mere clerk's question, but a question for every citizen who has a citizen's intellect as well as a private individual's needs.

**The Want of Money.**

"When I look back into the moderation of past ages," says Seneca, "it makes me ashamed to discourse as if poverty had need of any consolation; for we are now come to that degree of intemperance that a fair patrimony is too little for a meal. Homer had but one servant, Plato three, and Zeno, the master of the masculine sect of the Stoics, had none at all. The daughters of Scipio had their portions out of the common treasury, for their father left them not worth a penny." Moralising is dull work, hated by writers and readers alike. The most despised of all literature is sermons; therefore let no man who can do anything else publish sermons. Still, it is sometimes as interesting to know what people in earlier times thought of the practical worries and disagreeables of life and how they met them, as to try to reproduce the kind of coats they wore or the peculiar shape of the brass pans they warmed their beds with in cold weather. The want of money is in these days considered as next door to eternal perdition. By way of contrast, there is some consolation in the thought that Homer had but one servant. There may, indeed, have been times when he had none at all. He must be a vulgar and senseless animal who would not willingly live a whole lifetime without a servant if thereby he could leave the world such a legacy as Homer left it. It is undoubtedly trying to the moneyless man to find that all his other distinctions win him no honour among the multitude compared with the outward signs of respect which greet the man of millions. Besides, what wonders some of our clever fellows could perform if they had but "due furtherance of cash." Robinson, who writes the political articles for the *Mudborough Illuminator*, could get into Parliament, and might even be sent for by the Queen if the *Illuminator* could afford him two thousand a year instead of two hundred. But let poor Robinson be consoled. He has a maid-of-all-work to brush his boots and clean his wife's doorstep. We know on the authority of Seneca that Homer had no more. Many of us are glad and grateful that in one point at least we resemble the father of heroic verse. But, seriously, some of us have considerable reason to be ashamed of ourselves in that we blush for our want of wealth and meanly give place to Sir Gorgius Midas and his too often illiterate fellows. There are many things in the world beside wealth which are of first-rate value, and some of them much more valuable than wealth. To be well, to be honest, to have a family of healthy and well-conditioned children, to be esteemed and trusted by our intimates—these are advantages which wealth cannot always purchase. If all the world were rich to-morrow it would not be any more contented than it is now. A little philosophy and, still better, wholesomeness of disposition, make a good mixture for curing the mind of discontent.

**The Revolt of the General Practitioner.**

The *British Medical Journal* has shown itself the friend, not only of the struggling general practitioner, but of the whole medical profession in opening its columns freely for the discussion of the burning question of hospital abuse. There are signs, many and urgent, that the bulk of the profession are beginning to feel the pressure of hospital competition, and that those who feel it but a little are beginning to display an active and determined sympathy with those who feel it much. There are, moreover, unmistakable indications that the great body of practitioners will take up arms against hospital physicians and surgeons; and if they do, it will not be the general practitioner who will go to the wall. It ought to be understood by the public that at the present day there is no real distinction, so far as education and skill are concerned, between the competent general practitioner and the average hospital physician or surgeon. They have both passed through the same course of training, both faced and satisfied the same examiners, both taken the very same degrees or diplomas. In the ranks of general practice, as at the hospitals, there are distinguished graduates of every British university and professional college. What reason, therefore, can there be in the circumstance that whilst hospital physicians and surgeons rise higher and higher in public esteem and wealth, general practitioners should find themselves thrust lower and lower, at any rate in respect of the means of livelihood, until some of them find themselves distressingly near the gutter? There is no reason at all; and hospital physicians and surgeons are making the gravest possible mistake in separating their interests from those of the general practitioner. The interests of hospital physicians and ordinary practitioners are identical, not antagonistic. The medical profession is one, not two. Hospital physicians may affect to think there is no real grievance, but we know by indisputable evidence that there is a grievance of the most real and bitterly felt kind. If every London practitioner who feels keenly the competition of hospitals were to make known his grievance in the *British Medical Journal*, every page of that paper would be taken up by complaints, and there would be no space left for any other matter. There can be no doubt that all that is wanted is for hospital physicians to think of their outside brethren exactly as they would wish to be thought of if they were outsiders; and to do also as they would be done by. They, and they alone, have the power to mend matters; and they, and they alone, will be responsible if the general practitioner is driven by the stress of hospital competition into deeper despair and fiercer revolt.

